# Efficiency of 80% vs. 100% Oxygen for Preoxygenation: A Randomized Study on Duration of Apnoea Without Desaturation

**DOI:** 10.3390/jcm14217647

**Published:** 2025-10-28

**Authors:** Jaewoong Jung, Yang-Hoon Chung, Bon-Sung Koo, Sang-Hyun Kim, Hee-Chul Jin, Won Seok Chae

**Affiliations:** Department of Anaesthesiology and Pain Medicine, Soonchunhyang University Bucheon Hospital, Soonchunhyang University College of Medicine, Bucheon-Si 31538, Republic of Korea; 102978@schmc.ac.kr (J.J.); drcyh79@schmc.ac.kr (Y.-H.C.); kbs0803@schmc.ac.kr (B.-S.K.); skim@schmc.ac.kr (S.-H.K.); hcjin@schmc.ac.kr (H.-C.J.)

**Keywords:** patient safety, oxygen, hypoxemia, general anaesthesia, intraoperative monitoring

## Abstract

**Background/Objectives**: Preoxygenation with 100% oxygen is commonly used but poses risks such as hyperoxia and atelectasis. Using 80% oxygen may reduce these effects but shortens duration of apnoea without desaturation (DAWD). This study compared preoxygenation efficiency between 80% and 100% oxygen and evaluated changes in the Oxygen Reserve Index™ (ORi™). **Methods**: Patients undergoing elective laparoscopic cholecystectomy were randomized to preoxygenation with 80% or 100% oxygen. Adequate preoxygenation was defined as a ≤10% difference between fraction of inspired oxygen and end-tidal oxygen (EtCO_2_). The primary outcome was DAWD, the interval from apnoea onset to peripheral oxygen saturation (SpO_2_) of 93%. Secondary outcomes included time to adequate preoxygenation and additional warning time from ORi™ zero to SpO_2_ 97%. **Results**: Thirty patients were randomised to 80% (n = 15) or 100% oxygen (n = 15) oxygen groups. One patient in the 100% group was excluded due to spontaneous breathing before SpO_2_ 93%, leaving 29 for DAWD analysis. DAWD was 345 ± 136 s (80%) and 430 ± 163 s (100%) with a mean difference of 86 s (*p* = 0.135). No significant differences were observed in tie to adequate preoxygenation or additional warning time. **Conclusions**: Preoxygenation with 80% oxygen resulted in a numerically shorter DAWD compared with 100% oxygen, without a significant difference in ORi™. These findings may suggest the potential feasibility of using 80% oxygen for preoxygenation. However, given the limited sample size and uncertain clinical relevance, further large-scale studies are warranted to clarify the safety and clinical implications of lower oxygen concentration during anaesthesia induction.

## 1. Introduction

During anaesthesia induction, there is always the risk of unanticipated difficult mask ventilation or intubation [1]. To prevent arterial desaturation, a high inspired oxygen fraction (FiO_2_) is essential during preoxygenation. Traditionally, 100% oxygen has been the standard for preoxygenation, supported by various ventilatory techniques as tidal volume or vital capacity breathing [2].

A high FiO_2_ not only prolongs the duration of apnoea without desaturation (DAWD) but also reduces surgical site infection [3]. Nonetheless, prolonged use of 100% oxygen may lead to hyperoxia, increasing the formation of reactive oxygen species, which could cause pulmonary oxygen toxicity or vasoconstriction, especially in cerebral and coronary arteries [4]. Additionally, studies in the intensive care unit have shown that supplemental oxygen can lead to excessive hyperoxia, which is associated with increased mortality, emphasizing the importance of oxygen titration [5].

Although the duration of preoxygenation is brief enough to avoid hyperoxia-related complications, 100% oxygen carries potential risks such as atelectasis or delayed recognition of oesophageal intubation [6]. Atelectasis, mainly absorption atelectasis, is the most common potential issue following 100% oxygen administration during preoxygenation, Edmark et al. [7] demonstrated that 80% oxygen resulted in less atelectasis compared to 100% oxygen for 5 min during anaesthesia induction, and Akca et al. [8] found that the severity of atelectasis was comparable between patients receiving 80% and 30% oxygen during and 2 h after colon resection.

In clinical practice, anaesthesiologists prefer a FiO_2_ of ≥80% during anaesthesia induction [9]. Given the crucial need to balance preventing hypoxia and mitigating the potential risks of hyperoxia, demonstrating the efficiency of 80% oxygen in preventing desaturation is paramount. If there is no significant difference in DAWD between 100% and 80% oxygen, then 80% oxygen may be considered a viable alternative for preoxygenation. Therefore, this study aimed to compare the DAWD, a measure of the efficiency of preoxygenation, between 80% and 100% oxygen. Additionally, changes in the Oxygen Reserve Index (ORi™) (Masimo Corp., Irvine, CA, USA) were monitored during preoxygenation.

## 2. Materials and Methods

This single-centre, randomised trial was conducted at a regional tertiary hospital in South Korea. The study protocol was approved by the Institutional Review Board (IRB) of Soonchunhyang University Bucheon Hospital (IRB No. 2021-10-025) and was conducted in accordance with Consolidated Standards of Reporting Trial (CONSORT) statements. This study was registered with the Clinical Research Information Service of the Korea Centres for Disease Control and Prevention (KCT00007088).

### 2.1. Study Population

This study included patients undergoing elective laparoscopic cholecystectomy. Eligibility criteria included individuals aged 20–60 years, American Society of Anaesthesiologists (ASA) physical status of I-III without cardiopulmonary disease, and asymptomatic patients such as chronic cholecystitis or incidental gallstone or polyp not interfering with normal breathing. Exclusion criteria included a body mass index (BMI) > 35 kg/m^2^, inability to cooperate or claustrophobia, suspicious anatomical airway anomalies, and contraindications to the medication used during the study.

Patients provided informed consent the day before surgery and were briefed on the preoxygenation process. They were randomly assigned via web-based randomization program (www.randomization.com) to either the 80% oxygen group or the 100% oxygen group. The 80% group received 80% oxygen, while the 100% group received 100% oxygen during preoxygenation. To monitor FiO_2_ and end-tidal oxygen (EtO_2_) concentration during the procedure, only the patients were blinded to their group allocation.

### 2.2. Preoxygenation and Anaesthesia Protocol

All patients received an intramuscular injection of 0.2 mg glycopyrrolate as premedication. Upon entering the operating room, standard monitoring as recommended by the ASA was applied, including peripheral oxygen saturation (SpO_2_), electrocardiogram, and non-invasive blood pressure. A rainbow sensor^®^ (Masimo Corp., Irvine, CA, USA) was attached for ORi™ monitoring. Baseline vital signs were checked, and the proper function of all monitoring devices was confirmed before beginning preoxygenation.

Using the Avance CS2 (GE healthcare, Chalfont St Giles, UK) anaesthesia delivery system, the oxygen concentration for each group was maintained at 10 L·min^−1^ ≥ 5 min before the patient entered the operating room. A face mask was securely applied to prevent air leaks, and preoxygenation was initiated. Patients were instructed to breathe normally. Adequate preoxygenation was defined achieving a 10% difference between FiO_2_ and EtO_2_ [10]. During preoxygenation, end-tidal carbon dioxide (EtCO_2)_ graphs and values were monitored to ensure effective breathing, indicated by proper inflation and deflation of the reservoir bag.

**Figure 1:**
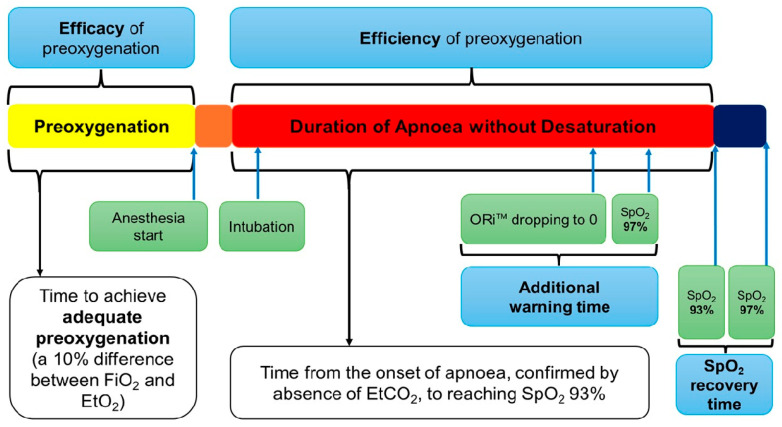
Study protocol. EtO_2_, end-tidal oxygen concentration; FiO_2_, fraction of inspired oxygen; ORi™, Oxygen Reserve Index™; SpO_2_, peripheral oxygen saturation.

Once adequate preoxygenation was confirmed, propofol (5 μg·mL^−1^ using the Marsh model) and remifentanil (2 ng·mL^−1^ using the Minto model) were administered for total intravenous anaesthesia. Upon observation of unresponsiveness to verbal commands, Train of Four (TOF) monitoring was initiated, and 1.5 mg·kg^−1^ of succinylcholine was administered. The onset of apnoea was confirmed by the absence of EtCO_2_ on the graphs. Following fasciculation and confirmation of a TOF of zero, endotracheal intubation was performed using a videoscope. If the vocal cords were not visible or endotracheal tube placement could not be visually confirmed, the patient was excluded from the study. After intubation, apnoea was maintained until SpO_2_ dropped to 93%. According to the British Thoracic Society, oxygen is used to maintain SpO_2_ levels between 94–98%, except in hypercapnic patients [11]. Therefore, the threshold was set at 93% SpO2 in this study. At SpO_2_ 93%, mechanical ventilation was initiated using each assigned oxygen concentration with a tidal volume of 6 mL·kg^−1^ and a respiratory rate of 10–12·min^−1^, and the time to reach SpO_2_ 98% was recorded. A schematic of the study protocol is illustrated in Figure 1.

### 2.3. Outcome Measures

The primary outcome was the time from the onset of apnoea to reaching SpO_2_ 93% after achieving adequate preoxygenation, which defined as the DAWD in this study.

Secondary outcomes included the time to achieve adequate preoxygenation, indicating the efficacy of preoxygenation, the maximum ORi™ value, the additional warning time, defined as the time from ORI™ dropping to zero to SpO_2_ decreasing to 97% [12], and SpO_2_ recovery time, defined as the time taken for SpO_2_ to increase from 93% to 98% after mechanical ventilation.

### 2.4. Statistical Analysis

Continuous data were assessed for a normal distribution using the Shapiro–Wilk test. Normally distributed data are presented as mean ± standard deviation and were analysed using Student’s *t*-test. Non-normally distributed data are presented as median and interquartile range (IQR [Q1, Q3]) and were analysed using the Mann–Whitney U test. Categorical data are expressed as frequency and percentages and were analysed using the chi-square test or Fisher’s exact test. A *p*-value < 0.05 was considered statistically significant. Statistical analysis was performed using IBM SPSS Statistics version 26 (IBM, Armonk, NY, USA) or Rex version 3.5.3 (RexSoft Inc., Seoul, Republic of Korea, http://rexsoft.org/ (accessed on 25 October 2025)).

### 2.5. Sample Size Calculation

The sample size was determined by an independent t-test comparing two means. Based on Edmark et al.’s study [7], where DAWD was 411 ± 84 s with 100% oxygen, the sample size was calculated with an alpha of 0.05 and beta of 0.1. A clinically meaningful difference in DAWD was predetermined at 105 s. This value was chosen because it represents the total intubation time using direct laryngoscopy in patients with a difficult airway [13], thereby as a clinical threshold. Based on these values, each group required 13 participants. Considering a 10% dropout rate, 15 patients per group were included, totalling 30 participants.

## 3. Results

**Figure 2:**
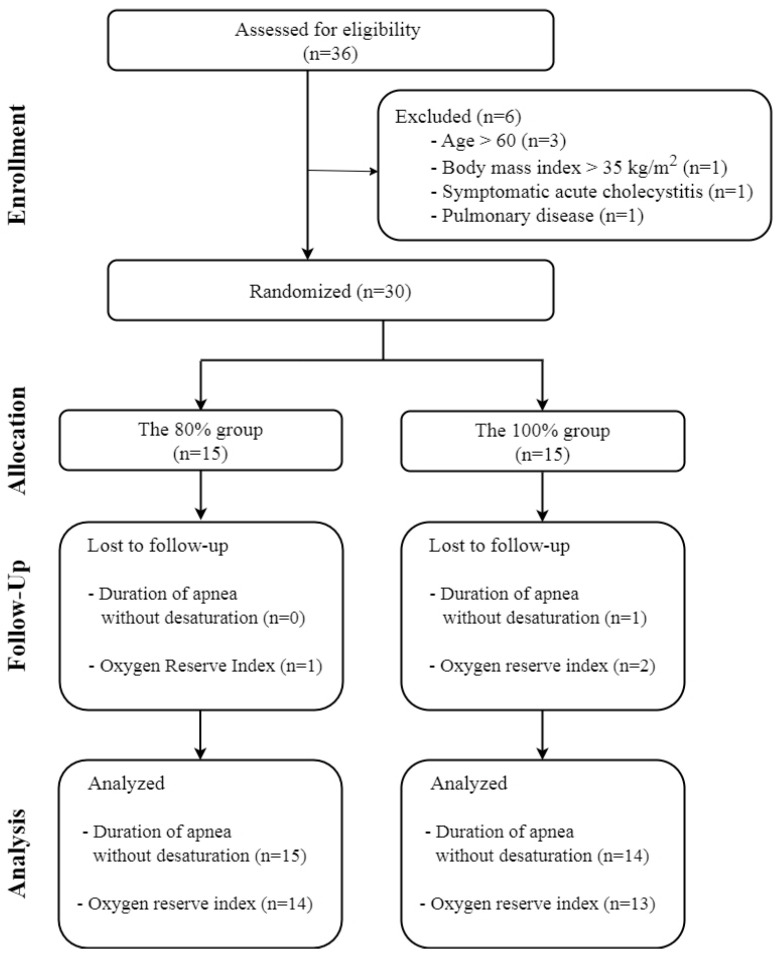
CONSORT flowchart.

From May to July 2022, 36 patients were enrolled in this study. However, six were subsequently excluded, including three with an age > 60 years, one with a BMI > kg·m^−2^, one with symptomatic acute cholecystitis, and one with pulmonary disease. Thirty patients were randomly assigned to the 80% group (n = 15) and the 100% group (n = 15). One patient recovered spontaneous respiration before reaching SpO_2_ 93% and was excluded from the analysis. Finally, the DAWD was analysed in 15 patients in the 80% group and 14 in the 100% group. ORi™ data were available for 14 patients in the 80% group and 13 in the 100% group due to recording errors (Figure 2). Demographic data of the patients are shown in Table 1.

### 3.1. Efficiency of Preoxygenation

The DAWD was 345 ± 136 s in the 80% group and 430 ± 163 s in the 100% group. Although the mean difference of 86 s between the groups was observed, it was not statistically significant (*p* = 0.135, 95% confidence interval [CI] −200 to 28 s).

### 3.2. Efficacy of Preoxygenation

The time required to adequate preoxygenation was 142 ± 53 s in the 80% group and 148 ± 58 s in the 100% group. There was no significant difference between the groups (*p* = 0.774, mean difference = −6 s, 95% CI −47 to 36 s) (Table 2).

### 3.3. Oxygen Reserve Index

**Figure 3:**
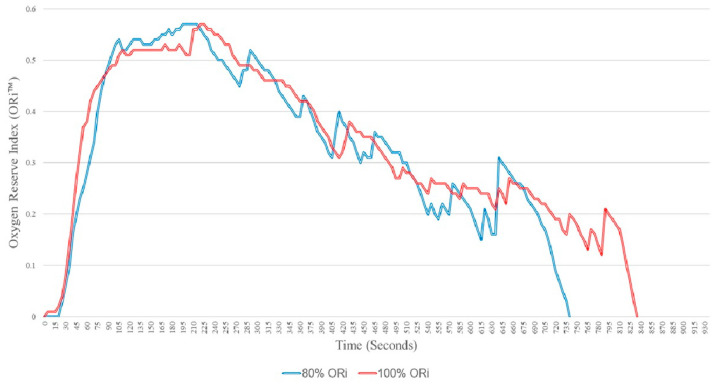
Changes in Oxygen reserve index (ORi™).

Changes in ORi™ are depicted in Figure 3, and ORi™ values are provided in Table 2. The time taken for the ORi™ to rise above 0 after oxygen administration was 49 ± 25 s in the 80% group and 42 ± 18 s in the 100% group, with no statistically significant difference (*p* = 0.430, mean difference = 6.5 s, 95% CI −10 to 23 s).

The time taken for the ORi™ to rise above 0 and then decrease back to 0 was 420 ± 132 s in the 80% group and 533 ± 182 s in the 100% group. Although the 100% group had a longer average duration of 113 s (95% CI −238 to 12 s), this difference was not statistically significant (*p* = 0.076).

The maximum ORi™ value was 0.61 ± 019 in the 80% group and 0.65 ± 0.31 in the 100% group, showing no significant difference between the two groups (*p* = 0.663, mean difference = −0.04, 95% CI −0.44 to 21.69).

The median additional warning time was 31 s (IQR [15.5, 45.5]) in the 80% group and 46 s (IQR [33, 84]) in the 100% group, with no statistically significant difference between the groups (*p* = 0.062).

### 3.4. SpO_2_ Recovery Time

The median time taken for SpO_2_ to recover from 93% to 98% was 37 s (IQR [34, 56]) in the 80% group and 41 s (IQR [35, 47]) in the 100% group. Despite the difference in FiO_2_, there was no significant difference in recovery time between the two groups (*p* = 0.844).

## 4. Discussion

This study aimed to evaluate the difference in DAWD when using 80% oxygen for preoxygenation compared to 100% oxygen, primarily by focusing on practical clinical indicators of the effectiveness of preoxygenation, rather than investigating the fundamental underlying physiology. Unlike the standard method of 3 min of tidal volume breathing, our study defined the endpoint for adequate preoxygenation using the difference between FiO_2_ and EtO_2_ reached 10%. This objective endpoint approach represents methodological advancement over time-based preoxygenation protocols providing a more standardized comparison between oxygen concentrations. Regarding the interpretation of the study results, this study was powered to detect a clinically significant difference of 105 s based on the time required for intubation in cases with a difficult airway. Therefore, our results should be interpreted with caution, as the observed mean difference of 86 s may have clinical relevance. However, considering the significantly shortened intubation times facilitated by video laryngoscopes compared to direct laryngoscopy [12,13], the 345 s of DAWD observed with 80% oxygen remains clinically significant, potentially providing sufficient time for anaesthesiologists to perform multiple intubation attempts. For example, in our previous retrospective study, the intubation time, monitored using EtCO_2_, was defined as the median time from the disappearance of EtCO_2_ before intubation to the appearance of the first EtCO_2_ after intubation, which was 41 s (IQR [35, 49]) [14]. Using 80% oxygen for preoxygenation in patients without cardiopulmonary disease provides 345 s of DAWD (allowing for approximately eight intubation attempts), a significant duration for anaesthesiologists.

Previous studies measuring DAWD have typically used 100% oxygen with techniques such as 3 min of tidal volume breathing or 4/8 deep breathing [15,16]. Edmark et al. [7] measured DAWD using 80% and 100% oxygen, reporting 303 ± 59 and 411 ± 34 s, respectively. Their preoxygenation technique was based on time rather than EtO_2_. In contrast, Bhatia et al. [17] aimed for EtO_2_ > 90% to achieve adequate preoxygenation in young adults with 100% oxygen, reporting a DAWD of 315 ± 100 s. Their method was similar to ours but differed in that they limited preoxygenation to 3 min, even if EtO_2_ had not reached 90%. The goal of preoxygenation is to increase the body’s oxygen reserves, where oxygen primarily stored in the lungs [2,6,18]. Monitoring EtO_2_ provides an objective, accurate, and non-invasive means of determining alveolar oxygen, which can be easily monitored in real time using anaesthesia machines in clinical practice; this makes it a practical endpoint for preoxygenation [10]. Therefore, using the 10% difference between FiO_2_ and EtO_2_ as a marker for adequate preoxygenation offers a simpler and more intuitive method for clinical application, resulting in a longer DAWD.

The efficacy of preoxygenation relates to the rate of oxygen wash-in in the lungs. In our study, there was no significant difference between the two groups in the time taken for adequate preoxygenation. This finding is consistent with our previous study [14], with nearly identical values between 80% and 100% groups. Although this result aligns with the physiologic law of exponential wash-in [19], our studies indicate that adequate preoxygenation can be achieved in a shorter time than the standard 3–5 min, suggesting that monitoring EtO_2_ can be clinically useful. However, it is worth noting that, in 27% of our participants (8 out of 30) a 10% difference between FiO_2_ and EtO_2_ was not reached within 3 min, as in our previous study [14].

ORi™, a relatively new monitoring tool, measures oxygen reserve status in the mild hyperoxia range (partial pressure of oxygen [PaO_2_], 101–200 mmHg) as a non-invasive, real-time technique. It provides values between 0 and 1. Despite individual differences [20] and variability even within the same individual when measured at different sites [21], changes in ORi™ values are significantly correlated with PaO_2_ [20,21]. Within the mild hyperoxia range, there is a strong positive correlation between ORi™ and PaO_2_, such that a decrease in Ori™ to zero can predict impending desaturation before a drop in SpO2 occurs [22]. This early warning capability is beneficial for obese patients [23] and high-risk surgical patients [24], making ORi™ a valuable tool for predicting impending desaturation in clinical settings. In the event of an unanticipated airway problem, recognizing the added warning time can enhance patient safety by allowing for rapid airway manipulation or calling for help before desaturation occurs. Monitoring ORi™ trend can provide anaesthesiologists with highly valuable information.

Our study has several limitations, Firstly, the sample size was calculated based on a previous study assuming a clinically significant DAWD difference of 105 s, which represents the approximate time required for a single intubation attempt during difficult airway management. Considering recent reports indicating a shorter successful intubation time in difficult airway due to advancements in videoscopes, it is plausible that a substantially larger sample size would have been required, Therefore, caution is warranted when interpreting these results, and their generalization is limited. Second, due to the inherent limitations of randomization, and likely exacerbated by the small sample size, there was demographic difference between the two groups. The 100% oxygen group was significantly (6.5 years) younger than the 80% oxygen group, which could have influenced the efficiency of preoxygenation. Since factors such as cardiac output or oxygen consumption, in addition to FiO_2_, can affect the efficiency of preoxygenation, further studies should control for theses variables. Lastly, although atelectasis prevention is a key potential benefit of 80% oxygen, its occurrence or severity was not directly assessed in this study. This absence limits our findings regarding the clinical benefits of 80% oxygen beyond DAWD. Thus, subsequent investigations employing objective methods, such as lung imaging techniques, are warranted to thoroughly assess and confirm the practical efficiency of 80% oxygen in mitigating atelectasis.

## 5. Conclusions

In conclusion, our study compared the DAWD following adequate preoxygenation with 80% versus 100% oxygen in patients without cardiopulmonary disease. Our findings indicated a numerically shorter DAWD (345 ± 136 s) with 80% oxygen compared with 100% oxygen. Additionally, ORi™ revealed no significant difference between the two concentrations. While these findings might suggest the potential utility of 80% oxygen for preoxygenation, such an interpretation is limited by the small sample size. Therefore, further large-scale studies are warranted to clarify the clinical implications and safety profile of lower FiO_2_ for preoxygenation, particularly to ensure patient safety during anaesthesia induction.

## Figures and Tables

**Table 1:** Patient demographic data.

	80% Group (n = 15)	100% Group (n = 15)	*p*-Value
Age (y)	46.1 ± 8.4	39.5 ± 9.0	0.049 ^a,^*
Sex (male: female)	5:10 (33.3%:66.7%)	8:7 (53.3%:46.7%)	0.269 ^b^
Weight	69.1 ± 15.8	72.7 ± 16.4	0.539 ^a^
Height	162.2 ± 10.8	167.5 ± 9.9	0.169 ^a^
BMI	26.2 ± 5.2	25.6 ± 4.3	0.766 ^a^
ASA (I:II:III)	7:7:1 (46.7%:46.7%:6.7%)	8:6:1 (53.3%:40.0%:6.7%)	1 ^c^
Haemoglobin	13.9 ± 1.8	13.7 ± 1.9	0.830 ^a^
Haematocrit	40.1 ± 5.1	40.4 ± 5.2	0.953 ^a^
Smoking (none: current: ex-)	12:2:1 (80%:13.3%:6.7%)	9:6:0 (60%:40%:0%)	0.215 ^c^

Data are presented as mean ± standard deviation or number (%). ^a^ Student’s *t*-test; ^b^ Chi-square test; ^c^ Fisher’s exact test; *, *p* < 0.05.

**Table 2:** Preoxygenation and Oxygen reserve index (ORi) data.

	80% Group	100% Group	*p*-Value	Mean Difference	95% CI
Preoxygenation					
Efficiency (Duration of apnoea without desaturation)	345 ± 136	430 ± 163	0.135 ^a^	−86 s	−200 to 28
Efficacy (time to achieve adequate preoxygenation)	142 ± 53	148 ± 58	0.774 ^a^	−6 s	−47 to 36
Time taken for adequate preoxygenation (≤180 s:>180 s)	12:3 (80%:20%)	10:5 (67%:33%)	0.682 ^b^		
ORi™					
Time from oxygen administration to first ORi™ > 0	49 ± 25	42 ± 18	0.430 ^a^	6.5 s	−10.3 to 23.4
Time from first ORi™ > 0 to ORi™ dropping to 0	420 ± 132	533 ± 182	0.076 ^a^	−113 s	−238 to 13
Maximum ORi value	0.61 ± 0.19	0.65 ± 0.31	0.663 ^a^	−0.04	−0.44 to 21.69
Additional warning time (from ORi™ dropping to 0 to SpO_2_ reducing to 97%	31 [15.5, 45.5]	46 [33.0, 84.0]	0.062 ^c^		

Data are presented as mean ± standard deviation, median [interquartile range], or number (%). ^a^ Student’s *t*-test; ^b^ Fisher’s exact test; ^c^ Mann–Whitney U-test.

## Data Availability

The raw data used in this study have not been posted to a public repository to preserve participant privacy and adhere to ethical standards. However, the data may be made available upon reasonable request to the corresponding author for research purposes.

## Abbreviations

DAWD	Duration of apnoea without desaturation
ORI™	Oxygen Reserve Index
EtO_2_	End-tidal oxygen
SpO_2_	Peripheral oxygen saturation
FiO_2_	Inspired oxygen fraction
IRB	Institutional Review Board
CONSORT	Consolidated Standards of Reporting Trials
ASA	American Society of Anaesthesiologist
BMI	Body mass index
EtCO_2_	End-tidal carbon dioxide
IQR	Interquartile range
CI	Confidence interval
